# An ‘instinct for learning’: the learning flights and walks of bees, wasps and ants from the 1850s to now

**DOI:** 10.1242/jeb.245278

**Published:** 2023-04-04

**Authors:** Thomas S. Collett, Natalie Hempel de Ibarra

**Affiliations:** ^1^ School of Life Sciences, University of Sussex, Brighton, BN1 9QG, UK; ^2^ Centre for Research in Animal Behaviour, Psychology, University of Exeter, Exeter, EX4 4QG, UK

**Keywords:** Insect learning, Insect navigation, Path integration, Insect brain, Neuroethology, Visual memories

## Abstract

The learning flights and walks of bees, wasps and ants are precisely coordinated movements that enable insects to memorise the visual surroundings of their nest or other significant places such as foraging sites. These movements occur on the first few occasions that an insect leaves its nest. They are of special interest because their discovery in the middle of the 19th century provided perhaps the first evidence that insects can learn and are not solely governed by instinct. Here, we recount the history of research on learning flights from their discovery to the present day. The first studies were conducted by skilled naturalists and then, over the following 50 years, by neuroethologists examining the insects’ learning behaviour in the context of experiments on insect navigation and its underlying neural mechanisms. The most important property of these movements is that insects repeatedly fixate their nest and look in other favoured directions, either in a preferred compass direction, such as North, or towards preferred objects close to the nest. Nest facing is accomplished through path integration. Memories of views along a favoured direction can later guide an insect's return to its nest. In some ant species, the favoured direction is adjusted to future foraging needs. These memories can then guide both the outward and homeward legs of a foraging trip. Current studies of central areas of the insect brain indicate what regions implement the behavioural manoeuvres underlying learning flights and the resulting visual memories.

## Teaser

**Summary:** Ancient Greek philosophers already recognised insects as intelligent. The more recent discovery of learning flights helps reveal how the interaction between instinctual and learnt behaviours structures this intelligence.

## Introduction

Learning and remembering one's visual surroundings well enough to navigate to significant places is a widespread skill among visually endowed species in vertebrate and invertebrate phyla, but in only a few species of ants, bees and wasps have we revealed the precise movements that enable an animal to acquire the necessary visual information. Hymenopterans have long been viewed as intelligent, but with little concern whether this intelligence is inborn or acquired through individual experience. One early explorer of the natural world was Albertus Magnus who, at the start of the 13th century, gave a reasoned account of why insects such as bees must have memories of places (see Box 1). It was the discovery of ‘locality studies’ or ‘orientation flights’ as they are often termed, that may have provided the first proof that insects learn.

In this Review, we start with research that led to this understanding. It began in the 1860s, half a century before Journal of Experimental Biology could have published the work, with an observation of solitary wasps leaving their nest in the ground. The wasps’ locality study was limited to the immediate surroundings of the nest and included phases in which the wasp looked back at it. Similar observations on a variety of solitary wasps and bees continued through the early decades of the 20th century. We retell these to celebrate the acute observations and interpretations of the skilled naturalists of that time.

We then jump to the last quarter of the 20th century, when video recording became widely accessible and allowed more detailed analyses of the learning flights of bees and wasps and the learning walks of ants. With video analysis it became possible to probe the details of the insects' visual ‘perception’ of their surroundings and what they remember about them. Thus, 150 years since locality studies were first observed, study of the precise movements of hymenopterans as they learn how to return to significant localities continues, but now informed by some understanding of the mechanisms of insect navigation.

We also chart how views of the ‘mind of the insect’ have changed over this time. In 1904, Auguste Forel, an anatomist, psychiatrist and outstanding myrmecologist, wrote in a pamphlet about ants that a ‘psychology that would ignore brain-activity is a monstrous impossibility’ (Forel, 1904). At about the same time, Cajal marvelled at the anatomy of the insect visual system, and later wrote: ‘the complexity of the insect retina is stupendous, disconcerting and without precedent in other animals […] the comparison of a rude wall clock with an exquisite […] watch fails to give an adequate idea of the contrast’ (Cajal, 1966). There is now a better understanding of what makes this visually engaged ‘watch’ tick. Indeed, we can relate some of the details of learning manoeuvres and the consequent visual memories to what is now known about the most significant central brain structures in arthropods: the central complex, which mediates learning manoeuvres, and the mushroom body, which houses memories.

**Box 1. The wisdom of Albertus Magnus**

The first experimental proof of insect memory may well have stemmed from the discovery of locality studies, but are there earlier discussions of insect memory? Our search stopped when we encountered Albertus Magnus. He was a Dominican friar born around 1200, who, whenever possible, given his ecclesiastical duties, pursued his many secular interests. He travelled widely, conversing and observing (Resnick and Kitchell, 2022). There are 38 volumes of his work that include theology, philosophy, zoology, botany, physiology, medicine, astronomy, astrology, geography, mineralogy and alchemy. His *De Animalibus* (translated by Kitchell and Resnick, 1999) is ∼2000 pages in length; the following quotations come from book 21, chapter 2:

*‘Some animals are perceived to have few or none of the interior powers, and some others are so much richer in these that they seem to have something akin to reason. Further still, of those which have virtues and powers of this sort, some have a certain subtlety in them (just like the bees) which others greater in size do not have. […] Memory occurs in some […] because memory is that which causes the sensible, in its absence, to return out of what was received earlier by sense, just as […] vultures once they are sated depart from the place of a cadaver and later return to it from the memory of the place and the cadaver… any animals which follow only a present sensible and do not return to the sensible when it is absent on the basis of the one received earlier have no memory of the things received earlier [... like] flies which, when they are driven away, fly back unmindful of a blow received earlier[…]. Indeed, memory may be defined as a treasure house of the forms received previously in sensation.[…] This is clear among bees and, likewise, ants, which have great prudence in collecting things and yet are not instructed. It happens, on account of this prudence, that they provide storehouses for themselves.’*

Albertus Magnus

This argument may be a distillation from Albertus' 20 years of study of Aristotle's scattered writings on memory. More recently, Connell (2021) has brought together Aristotle's thoughts on animal cognition.

## Henry Bates’ insight and its proof

Henry Bates, who travelled with Alfred Wallace to the Amazons, observed a locality study in a wasp leaving its nest and intuited its function: ‘I have said that the *Bembex* on leaving her mine [nest] took note of the locality […] a mental act of the same nature as that which takes place in ourselves […] The senses must be immeasurably more keen […] for to my eye there was no landmark on the surface of the sand which could serve as a guide […] The mind of the insect seems to be so constituted that the impression of external objects causes it to act […] like a machine constructed to move in a certain given way’ (Bates, 1863). Romanes, in his book *Animal Intelligence* (Romanes, 1883), offered a slightly different perspective: ‘It is essential that we should refer again to the observations of Messrs. Belt and Bates […] For it is evident that these sand wasps took definite pains to teach themselves the localities to which they desired to return’.

A crucial test of Bates' intuition came later from Niko Tinbergen (1932). He was interested in the kinds of visual landmarks that sand wasps relied upon for locating their nest. He placed pinecones in a circle around a wasp's nest, displacing them after the wasp's departure. The returning wasp searched for its nest relative to the displaced pinecones, indicating that its visual memory of the pinecones enabled it to find its nest. Tinbergen compared flat objects and those that stick up above the ground and found the latter to be more effective as landmarks. Forty years later, Tinbergen was engagingly modest about these famous experiments. ‘There is nothing much original in these papers. The Frenchmen Henri Fabre and Charles Ferton, the American Phillip Rau and the great Dutch naturalist Jac P. Thijisse had kindled my interest in digger wasps; Karl von Frisch's work had shown me the power of simple experiments in as natural conditions as possible; and Mathilde Hertz's work […] had inspired my first steps’ (Tinbergen, 1972). In fact, two other great American wasp enthusiasts, George and Elizabeth Peckham, report a simple experiment by Erneste Marchand, holidaying in Cursac, that predates Tinbergen's work (Peckham and Peckham, 1905). A wasp was misled to search for its nest by displacing a plant 2 feet from its position close to the nest, making the wasp on its return follow the plant and search 2 feet away from the nest. Marchand waited anxiously for the wasp to come back ‘I looked at my watch to see whether I could consecrate yet a few more minutes to curiosity without making my kind host wait too long. It was only half past eleven; we usually did not breakfast before noon [time passes]. My *Bembex* had a memory.’

Locality studies are not restricted to the nest. Thomas Belt (1874) in his book *The Naturalist in Nicaragua* relates how a wasp (*Polistes carnifex*) having caught a caterpillar too large to take home in one trip took note of the plant located near the remains of the caterpillar before leaving: ‘it hovered in front of it […] then took small circles in front of it, then larger ones around the whole plant’.

## Naturalists at the start of the 20th century

The Raus (Phil and Nellie) and the Peckhams each wrote books in the early 1900s with graphic accounts of the behaviour of wasps, reliving their countless hours of waiting for things to happen, during which they sometimes observed locality studies (Peckham and Peckham, 1905; Rau and Rau, 1918). Peckham and Peckham (1905) describe how the locality study of the wasp *Sphex ichneumonia* may depend on the complexity of the nest's surroundings: ‘she came out and walked slowly about in front of her nest and all around it. Then she rose and circled just above it, gradually widening her flight, now going further afield and now flying in and out among the plants and bushes in the immediate vicinity’. The wasp *Astata unicolor* differs in mostly walking in a complex path with few episodes of flight and returning often to the nest. Rau and Rau (1918) outline the more focused flight of the bee killing wasp, *Philanthus punctatus*: ‘we found one insect hovering over her nest […] performing her flight of orientation […] swinging to and fro, in semicircles with her head always toward the hole […] the arcs grew wider until suddenly she flew off’*.* They also marvel at behaviour of the bembicine wasp *Bicyrtes fasciata*: ‘She rises straight up in the air from her nest, so high that she is scarcely visible and then darts away. Her return is similar. She poises in mid-air 10 to 12 feet above the nest and drops straight down to land close to her burrow in a semi-barren sandy area’ (Rau and Rau, 1918).

Turner (1910) observed locality studies in honeybees leaving an artificial flower: ‘Facing the artifact and keeping about one cm from its surface, she would sidle, in a zigzag line, around the structure two or more times and occasionally re-enter it one or more times. Then she would describe one or more spirals, pausing at certain places in the environment as though examining landmarks. It seems plausible to interpret this as an act by which memory pictures of the environment are formed.’.

The variation between species is emphasised further by the locality study of the solitary bee *Osmia rufa*, which nests in holes in banks, trees or walls, always above the ground. In order to study the bee's behaviour, Balfour-Browne (1925) developed a technique of stacking artificial nest tubes – a method still used to encourage the nesting of these bees. He describes their locality study in his Royal Institution lectures: ‘Her next actions were with the object of learning the situation of the chosen [nesting] tube by flying backwards and forwards in front of it and facing it… [She] begins by a minute inspection of the entrance itself, the flight gradually extending on either side’.

These many accounts are of value. Some of the observed species may become rarer or even extinct. In this case, the verbal descriptions and rough diagrams of their locality studies may remain as the only evidence of a wasp's learning behaviour. Gould (1992; see also Gould and Marler, 1987) has a pithy suggestion that insect learning may be no more than ‘filling in blanks on a pre-printed form’. Applied to locality studies, the form is represented by the movements during the locality study (instinct) and the directed views so generated fill in the blanks (learning).

## New techniques for recording learning flights

**Fig. 1.**
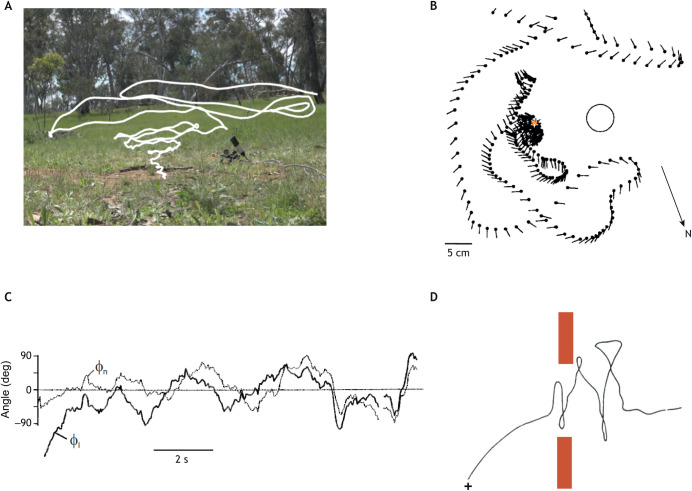
**Learning flights of *Cerceris* spp. and *Bombus terrestris***. (A) Low-resolution picture of a learning flight of *Cerceris australis* viewed using a video camera facing horizontally to capture the flight's increasing height and width. Image courtesy of J. Zeil. (B) Top view of the start of a flight of *Cerceris rybyensis*. Here and in later figures, the insect's position and body orientation on successive moments during a trajectory are shown by a stick (orientation) and ball (head). Orange asterisk indicates the nest; circle indicates a cylinder (landmark). (C) A wasp orientation relative to nest (φ_n_) and cylinder (φ_l_) during the flight in B. The wasp fixates its nest at the start of the flight. B and C are from Zeil et al. (1996). (D) An early depiction of a bumblebee (*Bombus terrestris*) learning flight. The bee left her nest (+) inside the laboratory and flew straight out of the open window. Only then did she reverse direction to fly in the typical zigzags of the bee's learning flight. Based on a drawing in Wagner (1907).

A big step in this truncated history was taken by Jochen Zeil, the major originator of contemporary studies of learning flights close to the nest. He exploited the easy availability of video recording to examine the details of the learning flights of the wasp *Cerceris* spp. (Fig. 1). One of his key findings is that the wasps faced the nest in the same direction on leaving the nest and on their return after foraging (Zeil, 1993a,b). This behaviour suggests that directed views memorised on departure guide a wasp's returns. The same is seen in bumblebees, where sometimes, as in wasps, the direction is specified through objects in the bee's close surroundings. More often and in different surroundings (a wooded garden and a bare open roof), the bee's preferred direction is governed by a compass and is to the north (Hempel de Ibarra et al., 2009).

Orientation flights cover a much larger area than a single video camera can capture. Visual observation gives some information about flights beyond the range of video, but real progress waited upon the development of harmonic radar by Joe Riley and colleagues (Riley and Smith, 2002). Radar responders attached to individual honeybees recorded the bees' horizontal flight position every few seconds over a distance of about 1 km from the hive. In its initial orientation flight, a honeybee flies a short distance from the nest and then comes straight back, covering a narrow sector around the nest. Over successive flights, the bee's speed and the length of its flight increase. Together, the flights explore in several directions (Capaldi et al., 2000). The efficacy of the flights was shown later. A honeybee, caught after its first orientation flight and released in a position along a previously explored direction from the hive, returned home more rapidly than when a bee was displaced to a point in an unexplored direction (Degen et al., 2016).

## Learning walks in ants and the significance of path integration

Because ants usually walk, rather than fly, their path and body orientation can be followed and recorded over relatively long distances. In recent years, their ‘learning walks’ have contributed greatly to understanding the behavioural mechanisms involved in place learning. Notably, the ant's behaviour during learning walks stresses the importance of path integration in fixating and learning nest-directed views, as Wehner and colleagues discovered when they observed the desert ant *Cataglyphis bicolor* leaving its nest for the first few times (Wehner et al., 2004).

Path integration is the ability of many animals on leaving a significant starting point to keep a running total of their distance and compass direction from that starting point (Mittelstaedt and Mittelstaedt, 1980; 1982; Müller and Wehner, 1988). It enables an animal to turn and face in the direction of a starting point, such as its nest, without any knowledge of the nest's visual surroundings.

**Fig. 2.**
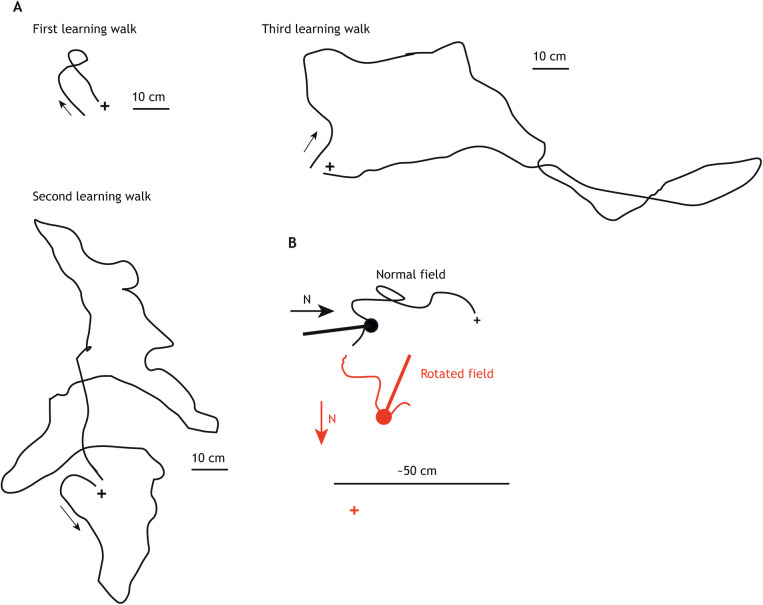
**Learning walks in ants.** (A) The first three excursions of *Cataglyphis bicolor* from its nest (+). The ant travels further from the nest with each excursion and then returns directly to the nest. The ant's direct path on its first excursion indicates that the homeward leg of the path is guided by path integration. Sketch based on Wehner et al. (2004). (B) Further evidence for path integration comes from rotating the magnetic field that the ant perceives. The ant follows the magnetic direction when fixating its nest (+) as shown by the nest-pointing direction of the coloured stick and ball. The nest and ant are coloured black when the applied magnetic field is off. The ant is then guided by the Earth's magnetic field. The ant is coloured red when the artificial magnetic field dominates and the ant faces a virtual nest (red +) indicated by the rotated field. Diagram based on data in Fleischmann et al. (2018).

*Cataglyphis bicolor*, on its first excursion from the nest, only travels a few centimetres before returning home (Fig. 2A). Successive excursions become longer; during these excursions, the ant periodically turns back to face the nest. Any disturbance of the ant by, for instance, an inadvertent movement of an observer makes the ant flee to its nest (Wehner et al., 2004).

Almost two decades later, the first indication that path integration is active during an ant's learning walk was supported by a more technically advanced study (Fig. 2B). Learning walks were recorded when an artificial magnetic field was applied to a small area around the nest. When the direction of the magnetic field was altered during an ant's learning walk, the ant faced the nest in the direction dictated by the magnetic field – proof that the nest direction was obtained through path integration rather than a visual memory of the nest's surroundings (Fleischmann et al., 2018). The use of a magnetic compass early in an ant's outdoor life is especially interesting. Unlike the more accurate sun compass, which needs to be calibrated to the local motion of the sun through the sky (e.g. Dyer and Dickinson, 1994), a magnetic compass needs no calibration.

**Fig. 3.**
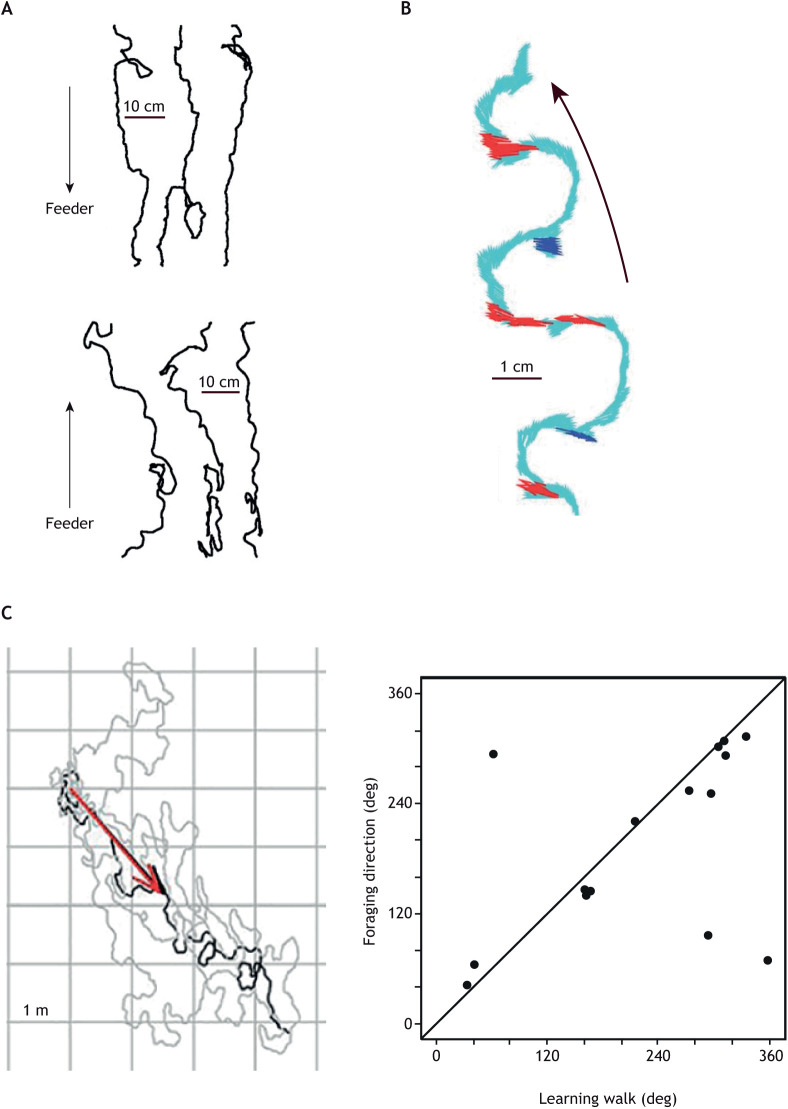
**Learning and foraging path directions of ants.** (A) When wood ants, *Formica rufa*, are first trained along a foraging route in the laboratory, they switch between looking in their future foraging direction and towards their starting point in both their initial outward and inward trajectories (which are marked by arrows). Image from Graham and Collett (2006). (B) Short excerpt from a learning walk of *Myrmecia croslandi* showing similar behaviour. The path is shown in light blue. The ant faces both towards its nest (red) and in the opposite direction (dark blue). Image from Jayatilaka et al. (2018). (C) Correlation between learning and subsequent foraging paths of *Melophorus bagoti*. Left: example of the last learning walk (grey) and first foraging trip (black). The thick overlapping black and red arrows show similar directions of the last learning walk and first foraging trip, respectively. Right: plot of foraging direction versus direction of the last learning walk for individual ants. Adapted from Deeti and Cheng (2021).

The ability of an observer to follow ants during their learning walks and later foraging trips makes it possible to see the relationships between the two. Inexperienced wood ants (*Formica rufa*) leaving a starting point along a foraging route (Graham and Collett, 2006) and bull ants (*Myrmecia croslandi*) first leaving their nest (Jayatilaka et al., 2018) tend to alternate between facing in the direction of a potential foraging route and facing towards their nest or starting point (Fig. 3A,B), suggesting that visual cues along the two directions are learnt together. New features of the relationship between learning walks and foraging emerge from the behaviour of *Melophorus bagoti* (Fig. 3C)*.* Not only are an individual's learning walks concentrated in the same direction in which the ant will later forage but also the set of directions chosen by a group of individual ants covers a large arc around the nest, enabling the group to explore the surroundings of the nest in many directions (Deeti and Cheng, 2021).

The way that wood ants perform learning walks indoors to remember the location of an inconspicuous sucrose feeder with its position marked by a cone and cylinder contributes to understanding how learning the image size of objects helps guide an ant's path. Contrary to expectation, ants do not fixate the sucrose. Instead, their strategy is to fixate one or other object, with the frequency of fixations decreasing as the ant's distance from the sucrose grows. The rate of decrease has the same profile, whether an object is placed 20 cm away from or immediately adjacent to the sucrose. This behaviour suggests that ants learn the image size of the cone and cylinder when at the feeder and monitor the objects' changing image size while moving away. Their knowledge of the landmarks' size at the feeder may explain why returning ants rarely face the location of the sucrose, moving instead towards one or other object (Nicholson et al., 1999).

## Facing the nest

A possible ecological difference is emerging in the time spent by different species in fixating their nest. *Cataglyphis fortis*, a desert ant which lives in barren surroundings, faces the nest briefly, whereas *Cataglyphis noda* and *Cataglyphis aenescens*, which inhabit pine forests in Greece, fixate the nest for longer (Fleischmann et al., 2017). Similar behavioural differences occur in the learning flights of wasps and bees. *Cerceris* spp., which tend to nest in bare sandy soil, fixate the nest briefly, whereas bumblebees, which often nest in undergrowth, have relatively long nest fixations (Robert et al., 2018; Collett et al., 2023).

The behaviour of bumblebees suggests that fixation length may be related to the value that bees assign to a goal. Bumblebees collect nectar and pollen from flowers. As we describe in more detail below, their learning flights after leaving a richly rewarding flower have longer fixations than those occurring after leaving a more meagre flower (Frasnelli et al., 2021). And fixations of the nest, which is probably of greater value to a bee than any flower, are longer still – though the flights may also be prolonged because nest holes are inconspicuous. This latter possibility gains support from the behaviour of insects after they have had difficulty in finding their nest. The learning flight on their subsequent departure from the nest is greatly prolonged (van Iersel and van den Assem, 1964).

## The contents of visual memories

What do insects learn about their surroundings during their learning flights and walks? This topic needs its own review and we only have space for a brief account. To answer this question properly, we must be sure that learning occurred on departure and not on approach. Miriam Lehrer (1993) tackled this problem with honeybees learning the location of a feeding site. She showed that the colours and shapes of objects can be learnt either on the bees' departure or on their arrival, but that knowledge of the distance of objects from the feeding site is only acquired on departure. Her method has not been adopted by others, so mostly we cannot say when a visual feature of a scene is learnt.

That honeybees can learn the angular separation of objects in an array relative to a food site was shown by spreading the array out and finding that the bees searched further from the array, thereby maintaining the angular separation between the objects (Cartwright and Collett, 1983). Similar experiments have been performed on hoverflies and desert ants, which find their hovering spot or nest by learning the apparent sizes of objects close to it (Collett and Land, 1975; Wehner and Räber, 1979).

**Fig. 4.**
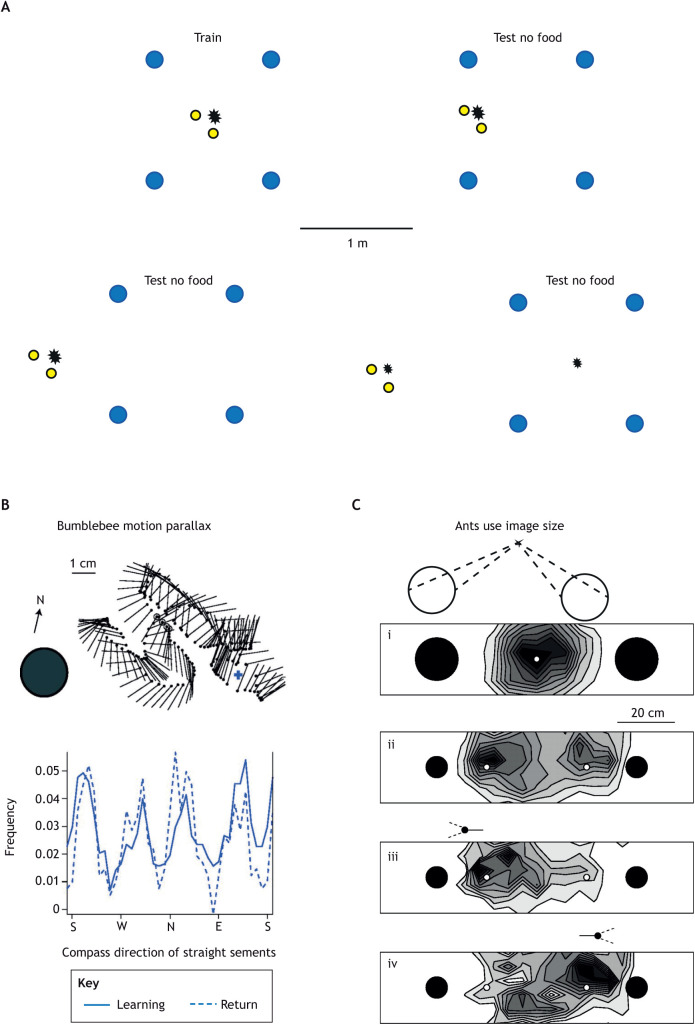
**How bees and ants measure the distance of visual objects from a goal.** (A) Honeybees learn the distance of a feeding site from the position of two narrow and short yellow cylinders close to the feeder and four larger blue cylinders further away. The asterisk indicates the centre of the bees' search relative to the cylinders when the feeder is absent. In tests, the yellow cylinders are moved further from the feeder site specified by the blue cylinders. Until this separation is ∼2 m, the bees search exclusively at the site indicated by the yellow cylinders. At ∼2 m, bees search in both possible sites. Redrawn based on data from Cheng et al. (1987). (B) Straight segments of flight that occur in bumblebee learning flights could, in principle, provide distance information through translational motion parallax. The frequency plot of the compass directions of the straight segments shows four equally spaced peaks which are similar across learning and return flights, suggesting similar usage of the segments during learning and returns. Modified from Collett et al. (2013). (C) Evidence that the ant *F. rufa* relies on the size of objects to guide its approach. (i) Ants are trained to find food midway between two cylinders. Dashed lines show the cylinders' image diameter when viewed from the food. When food is missing, ants search at the food site (position and density of search shown by intensity of shading). (ii–vi) Search distribution with smaller cylinders. (ii) Ants have two search sites: one close to each cylinder. (iii,iv) Data are partitioned. Ants facing the left-hand cylinder search to the left (iii). Ants facing the right-hand cylinder search on the right (iv). Reproduced from Graham et al. (2004).

Nonetheless, honeybees do encode the distance of objects from a feeder. For instance, given two arrays of objects with each array placed at a different distance from a sucrose source, trained honeybees can locate the position of the sucrose using either array (Fig. 4A). But if one array is shifted relative to the other, bees search preferentially in the position signalled by the array that is normally closer to the sucrose (Cheng et al., 1987), as in Tinbergen's experiment (Tinbergen, 1932).

Two cues to the distance of objects are widely available to insects: motion parallax and image size. Motion parallax is the perceived distance of surrounding objects viewed by a moving observer, with close objects seen to move faster than distant ones. This cue has been studied mostly in bees (e.g. Srinivasan et al., 1989; Dittmar et al., 2010). Bumblebee learning and return flights contain straight segments of flight. They are several centimetres long with compass directions roughly N, W, S or E (Collett et al., 2013) and could generate the required image motion (Fig. 4B). To use the retinal image size of an object as a cue to distance, an insect learns the object's image size when it is at a significant location, like its nest. It can then return there by moving until what it sees matches its memory. We illustrate the use of image size in wood ants which have learnt to find food midway between two cylinders (Fig. 4C).

So far, we have considered single places marked by nearby visual objects. Can honeybees avoid confusion, should they forage in two separate places with a similar arrangement of objects near the food, but with the food in each place positioned differently relative to the objects? The answer is yes. Collett and Kelber (1988) showed that when trained in this way, bees learn the broader spatial context of each food source, most probably relying on contextual cues to treat the two places as different. To minimise the chance that bees might learn the relationship between the array and other nearby objects, the two arrays were placed either in two identical huts or on two bare platforms in the open.

In natural surroundings, when landmarks are often viewed with the sky as background, insects with their UV receptors can take advantage of the strong UV contrast between the sky and objects on the ground beneath (Möller, 2002; Schultheiss et al., 2016). In addition to specifying location, visual scenes also indicate direction, as shown in Australian desert ants (Graham and Cheng, 2009) and honeybees (Towne et al., 2017). The ants (*Melophorus bagoti*) were trained to shuttle between their nest and a food site. Ants motivated to go home were placed in a 1 m diameter circular arena with a black wall shaped to copy the skyline. This artificial skyline was rotated relative to the normal homeward direction, causing the ants to head in the prescribed direction. Thus, a memorised scene along an insect's route can control its direction of travel.

How then are scenes remembered? Much of the relevant research relies on laboratory studies of ants or bees that have identified visual mechanisms like the ability to distinguish the orientation of edges (Srinivasan et al., 1993; Wehner, 1967), or to distinguish shapes such as a disc versus a triangle (Ronacher, 1998). In the latter case, changing the size of the disc or triangle did not disturb the bee's ability to identify the shapes. Much has been learnt about a wood ant's perception of shape by training an ant to approach a shape and then examining how it moves towards transformations of that shape (Lent et al., 2013). This procedure shows that ants learn and respond to both local properties of the shape (e.g. the orientation of an edge) and more global ones (e.g. the area or width of the shape).

## Matching learning and return flights

**Fig. 5.**
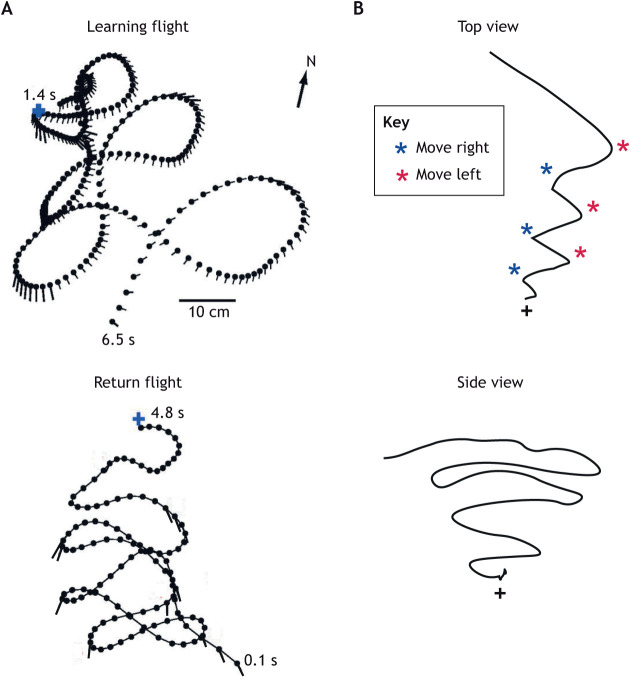
**Two ways in which return flights are guided by learning flights.** (A) Top: example of a sequence of loops during which the bumblebee *Bombus terrestris* faces the nest (+). The stick showing the bee's body orientation every 20 ms is lengthened when it fixates the nest within ±10 deg. Bottom: the same bee's zigzags on its return. The sticks just show nest fixations. Reproduced from Philippides et al. (2013). (B) Top: top view of the return flight of *Cerceris australis*. Blue asterisks indicate when the wasp recognises the memorised view of the nest (+) seen from the left; the wasp then turns right. Red asterisks indicate the opposite. Bottom: side view. This strategy leads to a cone-like flight path on the descent to the nest. Sketch based on Stürzl et al. (2016).

Nest facing during learning flights is often embedded within manoeuvres that are reflected in similar manoeuvres on the insect's return flight. Here, we examine two ways in which insects use information acquired during learning to aid their return (Fig. 5). Both *Cerceris* spp. and *Bombus terrestris* nest in holes in the ground. The learning flights of *Cerceris* start with the wasp leaving the nest and turning back to face it (Fig. 1C). The wasp then engages in a sequence of clockwise and anti-clockwise arcs of increasing height and radius that are centred on the nest (Fig. 1). The expanding arcs generate a cone of views of the nest. At the end of each arc, the nest is positioned about 30 deg to the left or to the right of the midline of the retina. By learning views of the nest from these points, a homing wasp encountering a memorised view of its nest on its way back (Fig. 5B) knows whether to move left or right in order to descend the cone (Stürzl et al., 2016).

The learning flight of *B. terrestris* starts with a period of several seconds in which the bee remains within about 5 or 6 cm of the nest hole and a few centimetres above it. The bee then gradually increases its distance from and its height above the nest (Linander et al., 2018). Bumblebees, in this later phase of the flight, perform a sequence of alternating clockwise and anti-clockwise loops, which carry the bees away from and towards the nest (Philippides et al., 2013). Returning bumblebees fly towards the nest, replicating the nest-approaching segments of the loops in a sequence of zigzags (Fig. 5A). Loops and zigzags are very similar within the range of ±50 deg of nest facing, so that the bee's approach on its return to the nest can be guided by the directed views memorised during the learning flight. Learning that is triggered when bees face the nest could be graded, being strongest at 0 deg and weakening as the nest is viewed more peripherally, thus helping returning bees to anticipate that they are about to face the nest.

## Learning flights from flowers

Flowers often advertise themselves by using bright colours and odours. They reward visiting insects and other pollinators by offering nectar and pollen. Honeybees on a single foraging trip tend to collect nectar or pollen from a sequence of flowers of the same species. This flower constancy benefits the bees because different flower species demand slightly different techniques for accessing the nectar. Darwin (1876) explained it thus: ‘The cause probably lies in insects being thus enabled to work quicker; they have just learned how to stand in the best position on the flower, and how far and in what direction to insert their proboscides.’. It took a century to have supporting evidence for this conjecture (Lewis, 1986). Perhaps a more significant reason for flower constancy is that bees learn the colour and shape of a visited flower and so can easily spot another flower of the same kind. This practice also benefits the plants, as the bees distribute pollen from one flower to fertilise another, so aiding outcrossing.

Bees learn the colours of artificial flowers both on their approach (Lehrer, 1993; Menzel, 1968; Opfinger, 1931) and on a learning flight on departure (Lehrer, 1993; Robert et al., 2018). Memories formed on departure after sampling the quality of the nectar are essential for a bee to know whether a particular flower is worth revisiting. In honeybees (Wei and Dyer, 2009) and bumblebees (Frasnelli et al., 2021), the length of their learning flights increases with the quality of the nectar (i.e. the sucrose concentration available in artificial flowers).

Bumblebee foraging differs from that of honeybees. Without a dance to tell other bees where to go for good flowers, bumblebees always make their own foraging decisions. These decisions depend in part on a bumblebee's size. Larger-sized individuals have larger eyes and more acute and sensitive vision than smaller-sized bumblebees (Spaethe and Chittka, 2003), and generally larger bees fly further and faster (Greenleaf et al., 2007). Their greater carrying capacity (Goulson et al., 2002) allows them to explore widely for good flowers and then take more nectar home. On leaving a flower, these larger bees match the length of their fixations of the flower to the quality of the sucrose that they have just drunk. Small bumblebees are less fussy and treat poorer and higher quality flowers in the same way (Frasnelli et al., 2021). Male bumblebees add an extra twist to this account. They leave their nest to live independently and sensibly do not perform learning flights on their departure. But in order to fend for themselves while finding queens, they must search for flowers and, after drinking from one, they perform a typical learning flight (Robert et al., 2017).

## Colour learning during learning flights

Colour learning in bees is historically interesting. It is a close contender to locality learning as one of the first proofs that insects can learn. Lubbock's work on colour learning (Lubbock, 1882) was championed by V. B. Wigglesworth (1976) and we cannot do better than let him describe it: ‘Since the time of Sprengel in the eighteenth century, it had been assumed that the colours of flowers served for the attraction of insects; and from this it was inferred that insects can distinguish colours. Lubbock set about testing the matter experimentally. He trained bees to visit honey smeared on pieces of glass which were laid on differently coloured papers. By moving the glasses around, he showed that bees always visited the colour to which they had been trained.’. Since then, increasingly sophisticated studies of colour learning in honeybees have refined our understanding (Hempel de Ibarra et al., 2014).

Wigglesworth went on to discuss Lubbock's view on learning and instinct: ‘Lubbock did not draw a sharp distinction between intelligent behaviour and instinctive behaviour. He did not divide animals into “little brain” types, rich in ready-made instincts but not susceptible to much education, and “big brain” types with few specialised instinctive capacities but with great powers of rapid learning.’. Nowadays, as Forel (1904) would have wished, the mechanisms underlying their behaviour can in part be understood by examining how the brain of an insect operates.

## Spatial learning in fruit flies and bees reveals much about the neurobiology of learning flights and walks

**Fig. 6.**
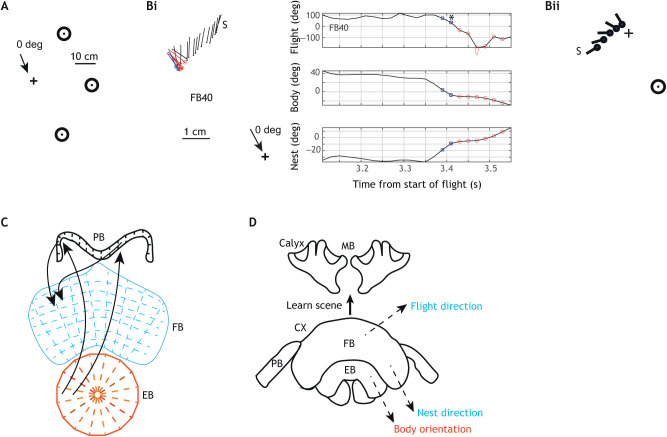
**Nest fixation during the first learning flight of the bumblebee, *Bombus terrestris.*** (A) Top view of the array of cylinders equidistant from the nest (+). Directions are relative to the arrow pointing from the nest to the bottom cylinder, which was the bumblebees' preferred body orientation in this experimental situation. (Bi) Events leading to the conjunction of nest fixation (±10 deg) and body at 0 deg (±10 deg). Left: segment of the flight trajectory of bee FB40. ‘S’ marks the start of a translational scan at about 90 deg leading to the conjunction of nest facing (red circles) and of body facing the bottom cylinder (blue squares). Right: time plots of flight direction, body orientation and orientation relative to nest. The asterisk shows when the bee first fixates the nest with body orientation at 0 deg. (ii) Schematic diagram of translational scan with the bee facing the nest until the body points at the bottom cylinder. A and B are from Collett et al. (2023). (C) The three major structures in the central complex (CX). Segments are separated by dashed lines. The ellipsoid body (EB) together with the protocerebral bridge (PB) encodes and transmits body orientation. The fan-shaped body (FB) contains circuitry mediating path integration and flight direction. Black arrows indicate some of the feedforward interconnections between compartments as shown in Fisher (2022). (D) Sketch of the bumblebee mushroom body (MB), which probably holds memories of visual scenes, and the CX, which mediates relevant flight parameters, and probably informs the MB when to learn a visual scene. The red and blue lettering of the flight parameters echoes the colouring of the CX structures in C that mediate them.

Understanding the mechanisms that underlie insect spatial learning relies upon information gleaned from a variety of insects. The ancestors of ants and flying hymenopterans diverged more than 350 million years ago (mya), yet have similar learning behaviour, probably underpinned by similar neural mechanisms. Crustaceans diverged even earlier from insects (400 mya) and have fundamental navigational mechanisms in common with insects (e.g. path integration; Zeil, 1998) and a central complex (CX; Fig. 6C,D) with the same basic divisions (Strausfeld, 2012). It has emerged more recently that the mushroom bodies (MBs) in the insect central brain (Fig. 6C,D) have analogous structures in crustacea (Wolff et al., 2017; Strausfeld et al., 2020).

The MB is crucial for learning olfactory and visual patterns. Strausfeld (2012) notes that Dujardin (1850), the first to study MB anatomy, also did behavioural experiments on honeybees, which suggested to him a possible role of this structure in the bees' intelligence (not necessarily learning). Evidence for a role in learning comes mostly from lesion studies. MB lesions in ants (Vowles, 1964) and honeybees (Erber et al., 1980) disrupt olfactory learning, and lesions in the MBs of ants prevent visual scene learning (Buehlmann et al., 2020; Kamhi et al., 2020). The importance of MBs in place learning was established in cockroaches through a ‘cat on a hot tin roof’ approach. MB lesions caused cockroaches to lose their memory of the location of a cool spot on a large hot plate (Mizunami et al., 1998). There is now evidence from desert ants that synaptic changes in the MB and CX occur during an ant's early visual exposure to the outside world and during learning walks (Groh and Rössler, 2020; Rössler et al., 2022).

**Fig. 7.**
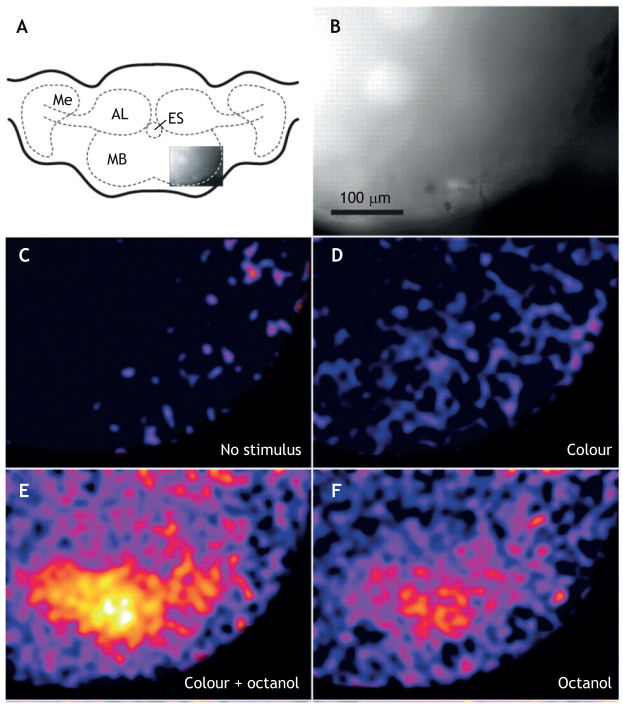
**Calcium activity in the MB calyx of the hawkmoth, *Manduca sexta*, after learning the colour and/or odour of a flower.** (A) A schematic illustration of the moth brain. Me, medulla; ES, oesophagus; MB, mushroom body; AL, antennal lobe. The inset indicates the position of the area enlarged in B. (B) Area of interest in the calyx where sensory input synapses with intrinsic cells of the MB. (C–F) Calcium activity in the area of interest indicated by the intensity of the yellow/red indicator. (C) In the absence of a stimulus. (D) With a colour stimulus alone. (E) Colour and odour stimuli. (F) Odour alone. Reproduced from Balkenius et al. (2009).

MBs receive olfactory and visual inputs in a variety of non-hymenopteran insects (e.g. cockroaches; Okada et al., 1999). The hawkmoth has been significant in uncovering the positive interactions that can occur between the two modalities. First, it was found that a combination of a flower's colour and scent is important in attracting these pollinators (Balkenius et al., 2006). Then, calcium imaging studies of the calyces of the MB (Balkenius et al., 2009) showed a striking enhancement in activity when the two modalities are co-active (Fig. 7).

The second crucial brain area involved in learning flights and walks – the CX – is primarily concerned with the coordination of movements. The functioning of its neural circuits is best understood in *Drosophila* through imaging circuit activity while tethered flies fly or walk. The role probably played by the CX in bumblebee learning flights can be illustrated by a bee's first fixation of the nest at the start of its first learning flight, when it has never seen the world outside its nest (Collett et al., 2023). The bee tends to face the nest (using path integration) while its body points at a favoured position in its surroundings. In this study, three black cylinders were arranged in an arc around the nest (Fig. 6A) and bees tended to point at the bottom cylinder when fixating the nest (Fig. 6Bi). Reaching the conjunction of facing the nest and the bottom cylinder is aided by a translational scan that is roughly perpendicular to the bee's pointing direction (Fig. 6Bi). During the scan, the bee can keep facing the bottom cylinder or the nest while the other variable reaches its preferred value (Fig. 6Bii).

Body orientation, nest facing and flight direction are controlled by the CX. In *Drosophila*, a fly's facing direction is encoded spatially within the ring-like ellipsoid body. The ring consists of 16 wedges. When the fly faces a point within a visual scene, activity within the ring is localised and rotates in synchrony with the fly (Seelig and Jayaraman, 2015; Hulse and Jayaraman, 2020). Visual input to the ring is carried by a population of ring neurons. Each neuron has inhibitory processes that connect all the pairs of wedges except one. Consequently, the active ring cell sets the point in the scene that attracts the fly's attention (Fisher et al., 2019; Kim et al., 2019). This circuitry would be well suited to enable a bee to face the bottom cylinder (Fig. 6A).

In bees, much of the circuitry supporting path integration resides within the fan-shaped body of the CX (Stone et al., 2017). The CX is also likely to control flight direction when the bee scans across the scene in order to face the nest and the bottom cylinder at the same time. In *Drosophila*, flight direction, independently of body orientation, is also computed within the fan-shaped body (Lu et al., 2022; Lyu et al., 2022). It is unclear how the CX might implement the coordinated manoeuvre of nest facing, body orientation and flight direction that leads to the appropriate conjunction of nest facing and body orientation (Fig. 6B).

## Conclusions and open questions

What contributions to our understanding of learning flights and walks have occurred over the last 50 years to enhance the insights of the earlier generations of naturalists? Perhaps the most fundamental advance is an understanding of the role that path integration plays in nest fixations. Video recordings have added details of learning flights close to the nest, and harmonic radar has uncovered what happens on a larger scale. These data suggest what visual information is acquired. The similarities observed between learning and return flights and walks of different species tell us the various ways in which these insects deploy the stored visual information to reach their nests and their foraging sites.

The most fundamental advances have come from neuroscientists working on other topics, such as the CX and MB of bees, ants and *Drosophila*. Consequently, the most interesting unanswered questions are concerned with the neural mechanisms that underlie the acquisition of visual information during learning flights and walks, and the changing properties of the memories acquired over a series of flights and walks. As almost always happens, interesting questions set technical hurdles. Much could be learned were it possible to record brain activity during learning flights or walks in tethered bees or ants. Fortunately, some progress has been made in this direction. Ant and bee navigational behaviour can now be viewed in tethered animals moving in virtual reality (e.g. Kócsi et al., 2020). Such studies can be valuable in themselves; for example, tethering ants that have been trained along a route makes it possible to examine the ants' turning behaviour when they are placed at different distances along and away from the route, thereby revealing interactions between path integration and visual route memories (Wystrach et al., 2020 preprint).

Perhaps an easier question to approach is whether visuo-spatial learning occurs in the optic lobes in addition to the MB? It is interesting because each layer of the optic lobes pictures the world as seen by the retina through a succession of processing layers (Strausfeld, 1976). But the connections between the optic lobes and the MB are probably not retinotopic. Parallel fibres in the lobes of the MB are contacted by visual inputs in the collar of the MB calyx (Gronenberg, 2001; Ehmer and Gronenberg, 2002; Fig. 6D). As specific visual features are viewed during fixations of the nest, potential benefits could come from modifying the responses of neurones in the optic lobes to emphasise the view seen during nest fixations.

It is foolhardy to predict where a field is heading. One side avenue might be behavioural experiments that probe notions of how the insect brain might operate. For instance, how does a bee's MB cope when it is asked to learn one view and then some time later another view that is similar to the first? The later view might displace the earlier one, both views might be learnt separately or an amalgam formed of the two. The MB’s solution may well differ according to the familiarity and use of the first view and how closely the views resemble each other. The only certainty is that new minds will find their own direction.
